# Underwater modified strip biopsy for colorectal polyp invading into the appendiceal orifice

**DOI:** 10.1016/j.vgie.2024.03.013

**Published:** 2024-03-26

**Authors:** Miyuki Iwasaki, Kenichiro Okimoto, Naoki Akizue, Yuki Ota, Takashi Taida, Tomoaki Matsumura, Jun Kato, Naoya Kato

**Affiliations:** Department of Gastroenterology, Graduate School of Medicine, Chiba University, Chiba, Japan

## Introduction

For colorectal EMR, polyps invading into the appendiceal orifice are one of the difficult cases.^1^^,^^2^ The location of the appendiceal orifice, the shape of the cecum, the thinness of the cecum wall, and fibrosis of the submucosal layer are also factors that make EMR difficult and increase the risk of accidental injury.^2^ Various endoscopic resections methods for polyps invaded into the appendiceal orifice have been reported.^1^ Recently, there have been some reports of endoscopic full thickness resection of polyps at the appendiceal orifice, but severe adverse events such as appendicitis, stricture, perforation, and bleeding have been reported.^3^ Eventually, there is no consensus on the treatment of such polyps. Here, we present the case in which such a polyp was resected via underwater modified strip biopsy (Video 1, available online at www.videogie.org).

## Case Presentation

**Figure 1:**
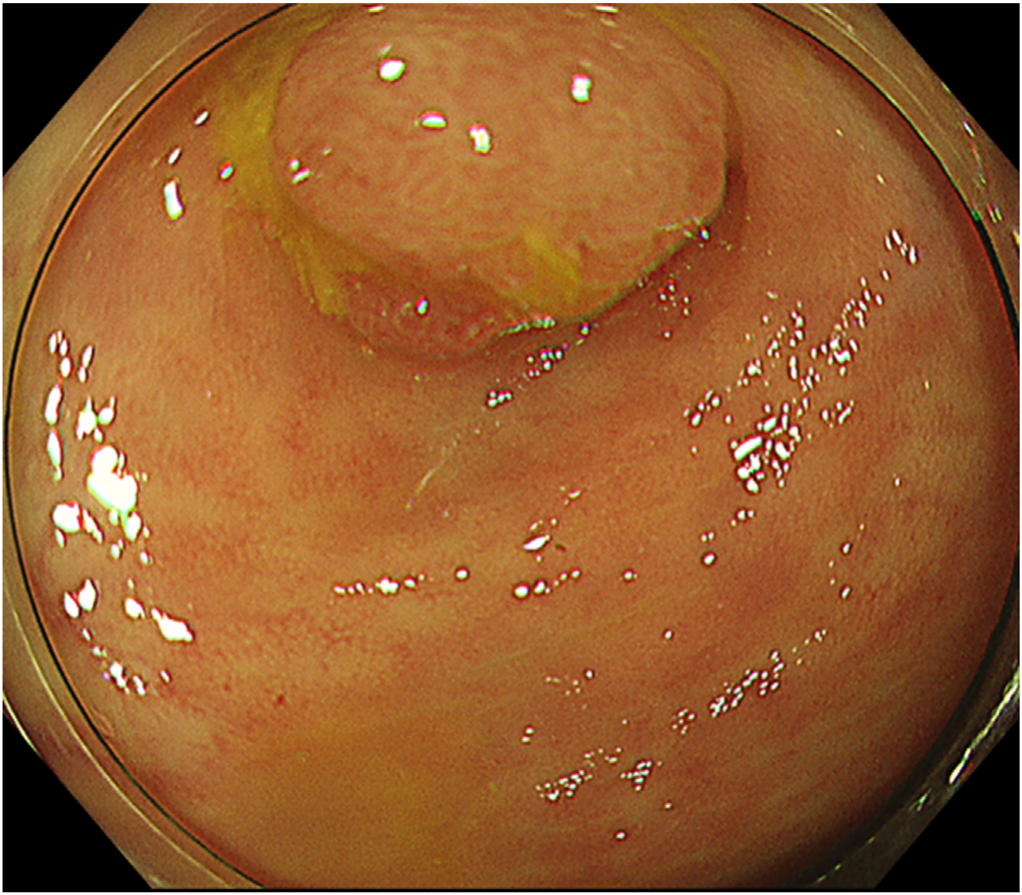
The polyp of the appendiceal orifice. White-light image of the polyp.

**Figure 2:**
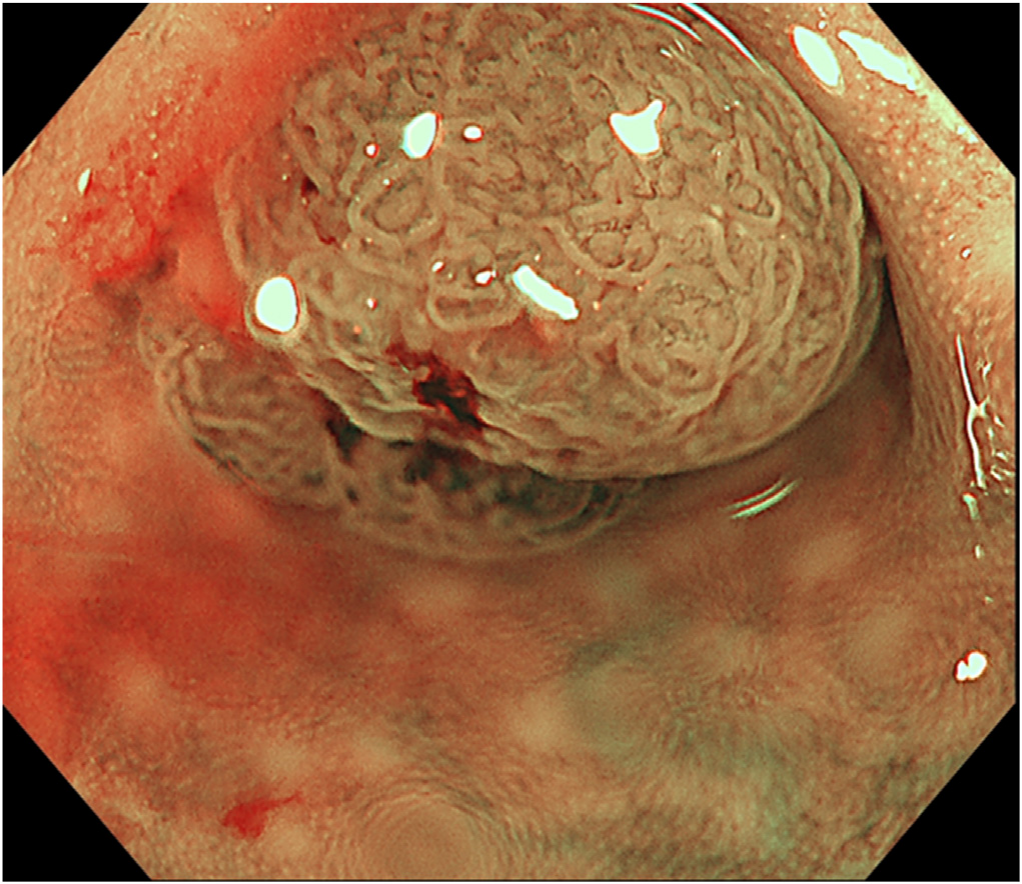
The polyp was classified with Japan NBI Expert Team type 2A suspected to be an adenoma.

**Figure 3:**
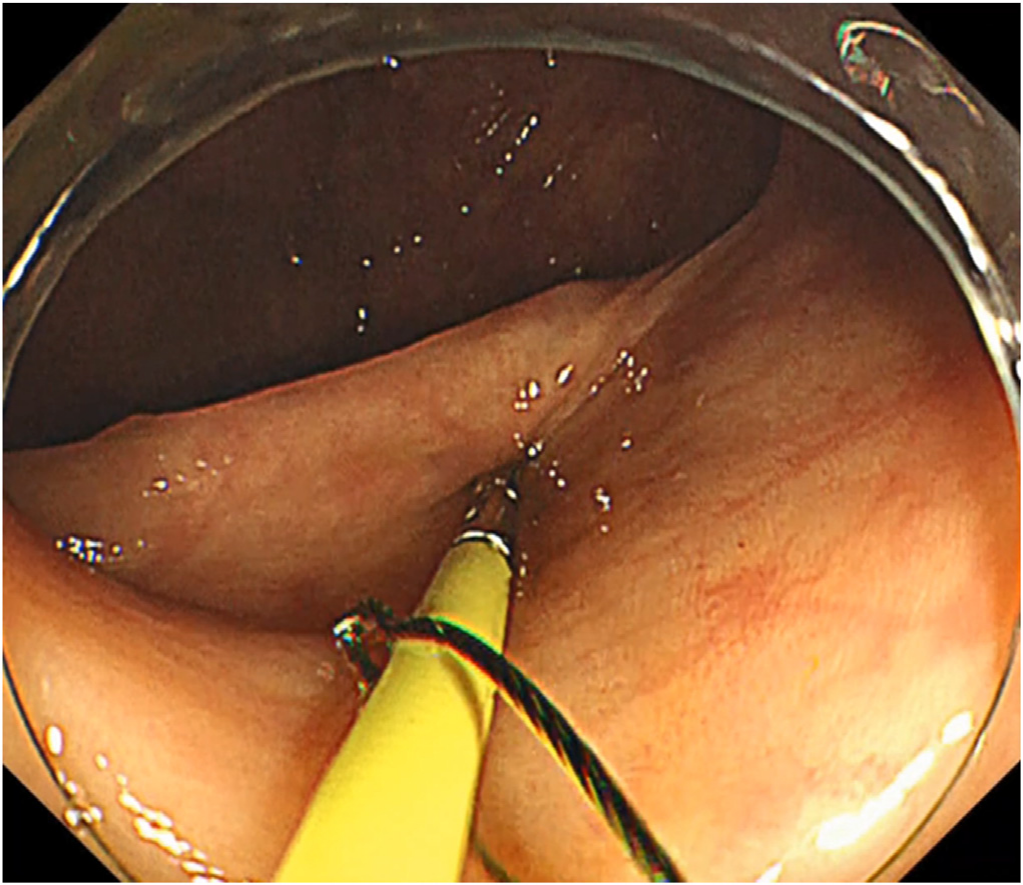
The procedure of underwater modified strip biopsy. The forceps was passed through the snare.

**Figure 4:**
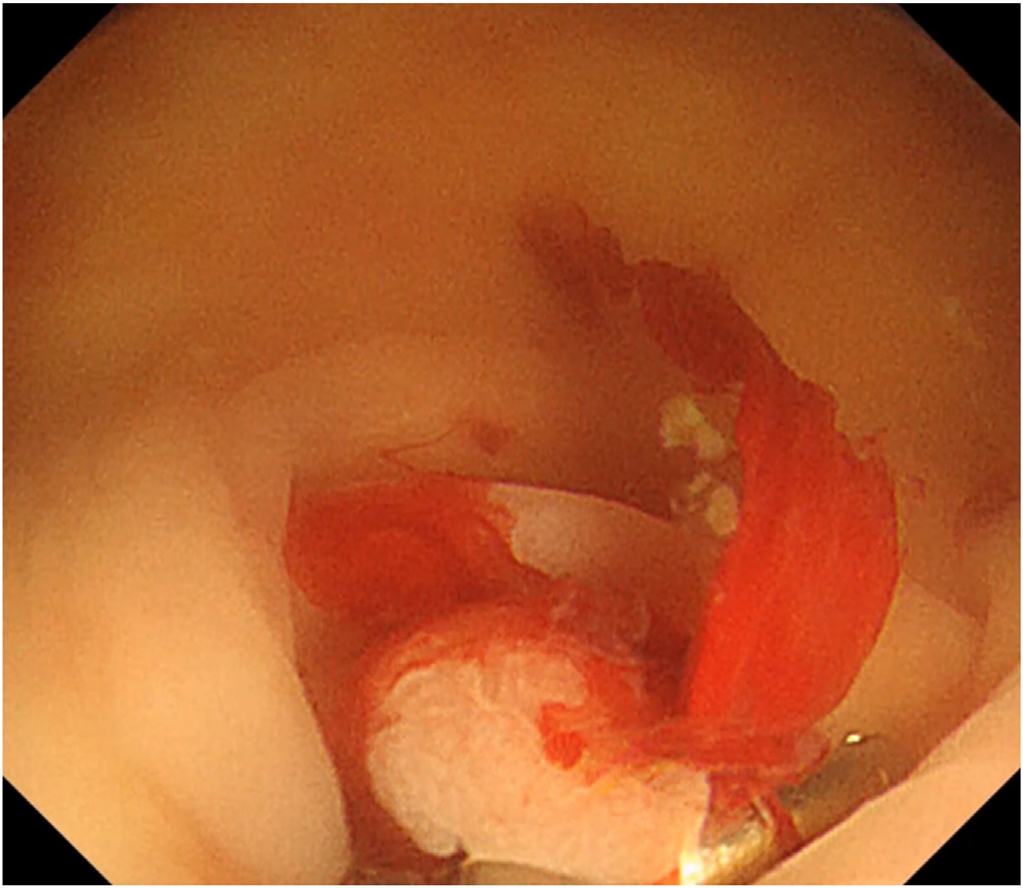
The polyp was grasped by forceps with underwater condition and was pulled out toward the lumen.

**Figure 5:**
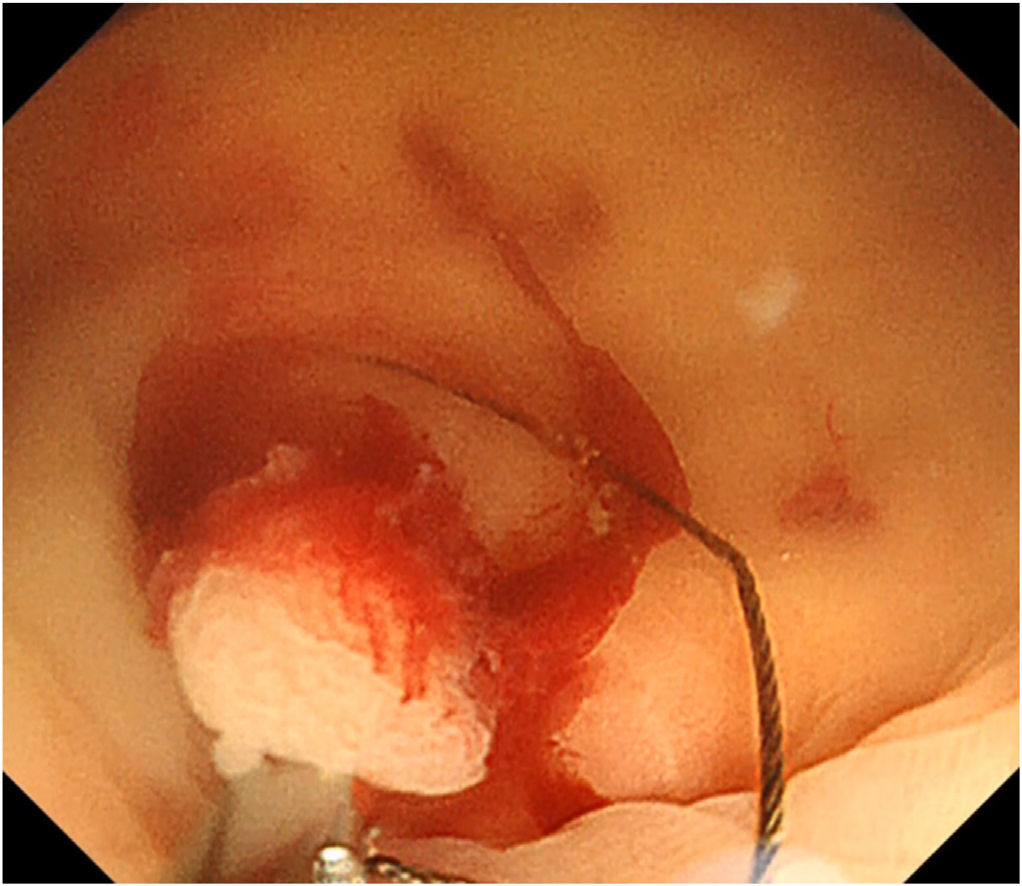
The polyp was resected by cold snare polypectomy.

**Figure 6:**
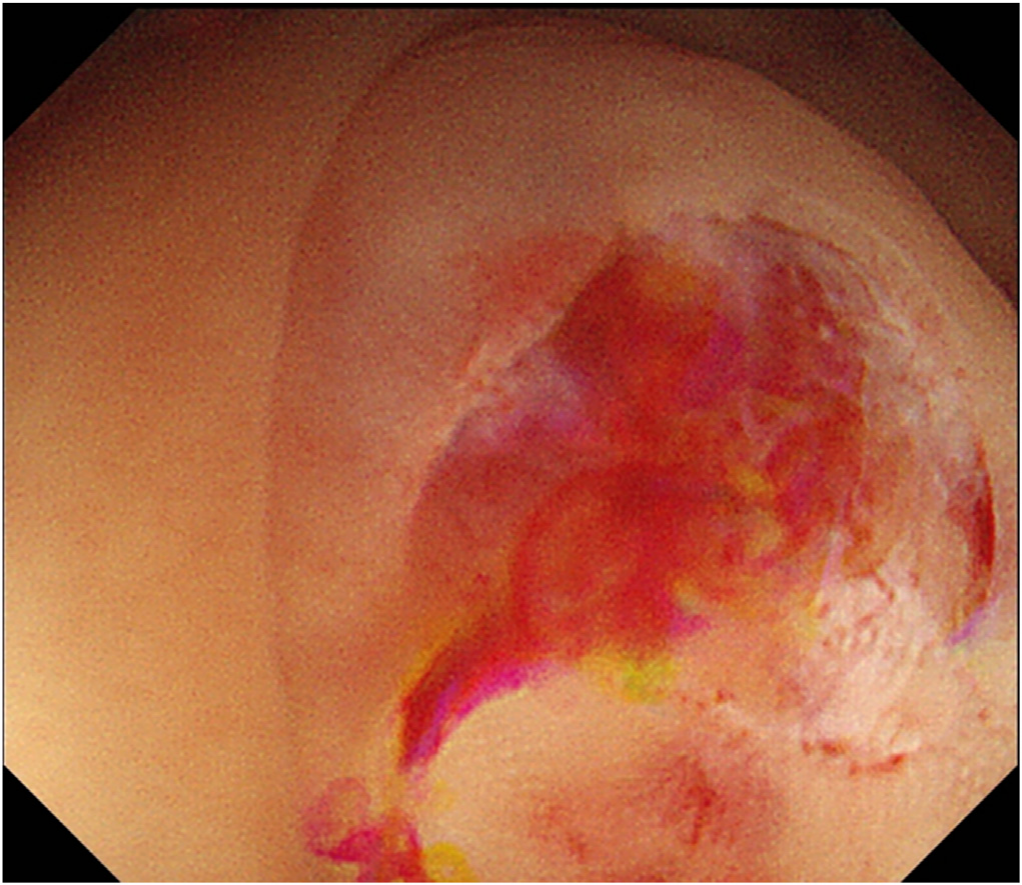
After resection, it was confirmed endoscopically that there was no residual lesion.

**Figure 7:**
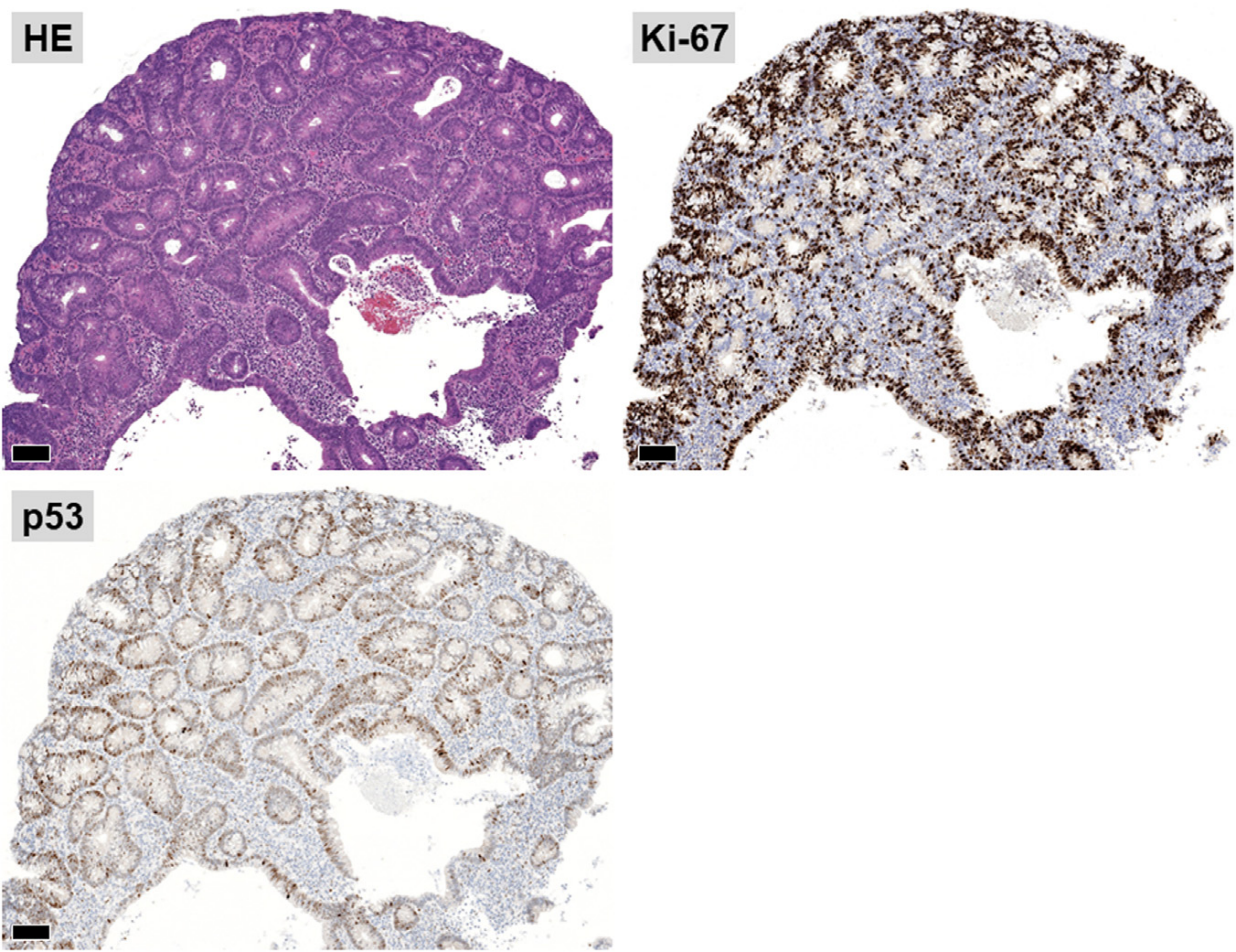
The pathology showed that the tumor was high-grade tubular adenoma, and the resection margins were unclear (pHMX, pVMX).

The patient was a 60-year-old man. The elevated 6-mm polyp invading into the appendiceal orifice was identified, but it was difficult to observe the entire polyp with CO_2_ insufflation (Fig. 1). The polyp showed Japan Narrow-Band Imaging (NBI) Expert Team type 2A, which was suspected to be an adenoma (Fig. 2). To observe the entire polyp supported by a strip biopsy technique, the procedure was performed using GIF-2TQ260M (Olympus Medical Systems, Tokyo, Japan), but it was still difficult to observe the entire polyp with CO_2_ insufflation. Therefore, we performed the procedure with underwater conditions using the floating effect. As the first step of the resection, the forceps (TechGrasper; MC Medical, Tokyo, Japan) was passed through the snare (AGS polypectomy Snare H; Hangzhou AGS MedTech Co, Ltd, Zhejiang, China; snare shape hexagonal, open width 10 mm) (Fig. 3). The polyp was observed and grasped by forceps with underwater condition, then pulled out toward the lumen (Fig. 4). Owing to the underwater condition, the polyp floated into the lumen and was easy to grasp and to confirm the polyp’s distal margin. The lesion was resected en bloc by cold snare polypectomy (CSP) (Fig. 5). the procedure time from passing forceps through the snare to resection is about 5 minutes. After resection, it was confirmed endoscopically that there was no residual tumor (Fig. 6). The pathology revealed the tumor to be a high-grade tubular adenoma (Fig. 7, scale bar 100 μm). The proliferative cell zone, positive for Ki-67, extended broadly from the superficial layers to the base of the glands, indicating enhanced proliferative activity. Conversely, p53 exhibited a wild-type pattern, with sporadic and discontinuous positive staining observed. The resection margins (both horizontal and vertical) were unclear. We scheduled follow-up magnifying endoscopy and biopsy from the resected scar 1 year after the treatment (at the time of the article, 1 year has not elapsed since the treatment).

## Discussion

GIF-2TQ260M is not widely available. It is a dual-channel, multi-bending scope available in Japan since July 2005. The scope has dual working channels and 2 bending sites (scope tip diameter 11.7  mm, channel diameter 3.2  mm/3.2  mm, working length 1030 mm). Although the endoscope is usually for upper GI tract, total colonoscopy was considered possible at the time of previous examination. Strip biopsy was originally a method in which normal saline is injected into the submucosa, which is then fixed with grasping forceps and resected by snare with an electric current.^4^^,^^5^ In this case, in addition to the advantage of the floating effect with underwater condition^6^ to confirm margin, the original strip biopsy was modified in that CSP was applied. Although endoscopic submucosal dissection and endoscopic full-thickness resection, including full-thickness resection device,^7^ could be other alternatives for polyps at the appendiceal orifice, safer en bloc resection is preferable, especially for small or no malignant lesions. Surgical resection including appendectomy with limited cecal resection is also too invasive for those lesions. As the safety of CSP has been demonstrated in several meta-analyses,^8^^,^^9^ forceps traction allowing a cold snare to be used was thought to contribute to reducing the risk such as perforation in this particular case. We have reported that underwater CSP for diminutive and small colorectal adenomas was safe and effective from the perspective of pathological complete resection.^10^ Using the floating effect under underwater conditions and using the strip biopsy technique can facilitate the elevation of lesions. As a result, it became easier to visualize the margins. This, in turn, contributed to a more reliable en bloc resection.

## Conclusions

Underwater modified strip biopsy could be one of the options for the resection of colorectal polyp invading into the appendiceal orifice.

## Disclosure

The authors disclosed no financial relationships relevant to this publication.

## Supplementary data

Video 1 The underwater modified strip biopsy.
